# Differential virulence and tsetse fly transmissibility of *Trypanosoma congolense* and *Trypanosoma brucei* strains

**DOI:** 10.4102/ojvr.v84i1.1412

**Published:** 2017-06-27

**Authors:** Purity K. Gitonga, Kariuki Ndung’u, Grace A. Murilla, Paul C. Thande, Florence N. Wamwiri, Joanna E. Auma, Geoffrey N. Ngae, James K. Kibugu, Richard Kurgat, John K. Thuita

**Affiliations:** 1 Kenya Agricultural and Livestock Research Organization – Biotechnology Research Institute (KALRO-BioRI), Kikuyu, Kenya; 2 Kenya Food Crop Research Institute, Nairobi, Kenya

## Abstract

African animal trypanosomiasis causes significant economic losses in sub-Saharan African countries because of livestock mortalities and reduced productivity. Trypanosomes, the causative agents, are transmitted by tsetse flies (*Glossina* spp.). In the current study, we compared and contrasted the virulence characteristics of five *Trypanosoma congolense* and *Trypanosoma brucei* isolates using groups of Swiss white mice (*n* = 6). We further determined the vectorial capacity of *Glossina pallidipes*, for each of the trypanosome isolates. Results showed that the overall pre-patent (PP) periods were 8.4 ± 0.9 (range, 4–11) and 4.5 ± 0.2 (range, 4–6) for *T. congolense* and *T. brucei* isolates, respectively (*p* < 0.01). Despite the longer mean PP, *T. congolense*–infected mice exhibited a significantly (*p* < 0.05) shorter survival time than *T. brucei*–infected mice, indicating greater virulence. Differences were also noted among the individual isolates with *T. congolense* KETRI 2909 causing the most acute infection of the entire group with a mean ± standard error survival time of 9 ± 2.1 days. Survival time of infected tsetse flies and the proportion with mature infections at 30 days post-exposure to the infective blood meals varied among isolates, with subacute infection–causing *T. congolense* EATRO 1829 and chronic infection–causing *T. brucei* EATRO 2267 isolates showing the highest mature infection rates of 38.5% and 23.1%, respectively. Therefore, our study provides further evidence of occurrence of differences in virulence and transmissibility of eastern African trypanosome strains and has identified two, *T. congolense* EATRO 1829 and *T. brucei* EATRO 2267, as suitable for tsetse infectivity and transmissibility experiments.

## Introduction

African animal trypanosomiasis (AAT) is a major cause of food insecurity, rural poverty and economic losses in the affected area of about 10 million km^2^ in sub-Saharan Africa (Ilemobade 2009). The disease is caused by protozoan pathogens of the genus *Trypanosoma*, a majority of which are cyclically transmitted by tsetse flies (*Glossina* spp.). The main economically important species in cattle, sheep and goats are the tsetse fly–transmitted *Trypanosoma congolense, Trypanosoma vivax* and *Trypanosoma brucei* spp. *T. vivax* has also been reported to be mechanically transmitted by tabanids and stomoxes (Desquesnes & Dia 2004). Other economically important AAT pathogens include *Trypanosoma evansi*, which is the main agent responsible for the disease in camels and is mechanically transmitted by biting flies (Desquesnes et al. 2013), and *Trypanosoma equperdum*, which causes dourine in horses and other equids and is transmitted through coitus (Brun, Hecker & Lun 1998). Upon ingestion of an infected blood meal by the tsetse fly, the cyclically transmitted trypanosomes establish in the midgut, undergo multiplication and differentiation and, subsequently, migrate anteriorly to the salivary glands (*T. brucei* spp.) or the mouthparts (*T. congolense* and *T. vivax*) where they mature into infective metacyclic forms (Matthews 2005). Infection of a new mammalian host begins when the metacyclic trypanosomes are intradermally injected through a tsetse bite (Stijlemans et al. 2016). In the mammalian host, metacyclics change their restricted repertoire of variant surface glycoproteins genes to the more elaborate system characteristic of bloodstream forms (BSFs) (David & McCulloch 2001). The mammalian host’s humoral immune response serves in selecting against the predominant BSF variants at any one peak of parasitaemia, thus giving rise to successive parasitaemic waves and enhancing the chances of transmission to new hosts.

*Trypanosoma congolense* is one of the most widespread livestock infective trypanosomes in tropical Africa found in ruminants, pigs, dogs and other domestic animals throughout the tsetse belt (Stephen 1986). *Trypanosoma brucei* spp. have a comparable host range and spatial distribution (Duffy et al. 2013). However, the prevalence and severity of clinical *T. congolense* infections in cattle has been reported to be higher than that of *T. brucei* spp. (Desta, Beyene & Haile 2013; Majekodunmi et al. 2013). These differences have been attributed to host susceptibility, intrinsic differences in trypanosome virulence and the vectorial capacity of vector tsetse flies for respective parasites. However, there is paucity of unequivocal experimental data to verify these claims. The current study was designed to investigate phenotypic differences in virulence of *T. congolense* and *T. brucei* isolates that were isolated from eastern Africa and are currently preserved at the Kenya Agricultural and Livestock Research Organization–Biotechnology Research Institute (KALRO-BioRI) cryobank. The vectorial capacity of *Glossina pallidipes*, the most economically important fly species in Kenya, for these animal pathogenic trypanosomes was thereafter investigated in order to assess the role of trypanosome virulence on transmission dynamics in the field and identify suitable isolates for laboratory-based tsetse infectivity and transmissibility studies.

## Methods

### Mice

Male Swiss white mice weighing 20 g – 30 g were obtained from KALRO-BioRI Small Animal Breeding Unit and used in this study. The animals were maintained at room temperature and fed on mice pellets (Unga Feeds Ltd, Kenya). Water was provided ad libitum, and wood chippings were used as bedding material. They were acclimatised for 7 days before the experiments commenced.

#### Tsetse flies

One thousand and fifty, 0- to 3-day-old teneral male *G. pallidipes* flies from the KALRO-BioRI tsetse colony were used. This colony was founded in the late 1990s with pupae obtained from the International Atomic Energy Agency Seibersdorf colony, which in turn originated from Tororo, Uganda (Ciosi, Masiga & Cmr 2014). The KALRO-BioRI laboratory colony is housed in an insectary maintained at 24 °C ± 1 °C and 75% ± 5% relative humidity and fed on defibrinated bovine blood every 48 hours through an artificial membrane feeding system (Feldmann 1994).

#### Trypanosomes

Trypanosomes stabilates were randomly selected from isolates that are currently cryopreserved at the KALRO-BioRI Trypanosome bank. The isolates included five *T. congolense* and five *T. brucei* spp. (Table 1); at the time they were used for the current study, the presumptive identification of these isolates as *T. congolense* or *T. brucei* spp. was based exclusively on morphological characteristics at the time of isolation. Upon the initial isolation, each of the isolates was multiplied in irradiated or immunosuppressed donor mice and at the first peak of parasitaemia, trypanosomes were cryopreserved as previously described by Murilla et al. (2014). Trypanosome isolates used for the current experiment had undergone a minimal 1–5 passage (Table 1) since isolation indicating that the essential attributes remain intact.

**TABLE 1:** Trypanosome species, locality, host and year of isolation.

Stab number	Locality	Species	Isolation year	Host of isolation	Number of passages
KETRI 2909	Galana, Kenya	*Trypanosoma congolense*	1983	Bovine	1
KETRI 2773	Galana, Kenya	*Trypanosoma congolense*	1984	Bovine	5
KETRI 2784	Matuga, Kenya	*Trypanosoma congolense*	1981	Bovine	1
EATRO 2254	Lugala, Uganda	*Trypanosoma congolense*	1976	Bovine	1
EATRO 1829	Ikoma, Tanzania	*Trypanosoma congolense*	1970	Bovine	1
EATRO 2267	Lugala, Uganda	*Trypanosoma brucei* spp.	1976	Bovine	1
EATRO 2225	Kagezi, Tanzania	*Trypanosoma brucei* spp.	1974	Bovine	1
EATRO 1579	Otuok, L. Valley	*Trypanosoma brucei* spp.	1970	Bovine	3
EATRO 1784	Otuok, L. Valley	*Trypanosoma brucei* spp.	1970	Tsetse fly	2
KETRI 2795	Matuga, Kenya	*Trypanosoma brucei* spp.	1981	Sheep	1

#### Virulence studies

The trypanosome stabilates were suspended in phosphate saline glucose, pH 8.0 and intraperitoneally (IP) inoculated into two donor Swiss white mice per stabilate; the mice had, before infection, been immunosuppressed using cyclophosphamide administered IP at 100 mg/kg per day for 3 consecutive days as previously described (Wu et al. 2015). When these donor mice attained peak parasitaemia (approximately antilog 8.1), they were deeply anaesthetised and bled from the heart and subsequently euthanised. The harvested blood (approximately 1 mL) was placed in tubes containing ethylenediaminetetra-acetic acid (1.5 mg/mL) for quantification and serial dilution of parasite numbers to provide an inoculation suspension of 1 × 10^4^ trypanosomes per mouse contained in 0.2 mL of the suspension as previously described (Thuita et al. 2008). All the experimental groups consisting of six Swiss white mice per trypanosome isolate were IP inoculated with 1 × 10^4^ trypansomes. Six mice were used as non-infected (negative) controls. Post-infection monitoring was carried out for a total of 30 days after which any surviving mice were euthanised and their survival time categorised as censored data. The infected mice were monitored for the onset (pre-patent [PP] period) and level of parasitaemia using the matching method (Herbert & Lumsden 1976). Packed cell volume (PCV) was monitored weekly using the method outlined by Naessens et al. (2005) while body weight changes were also monitored weekly using an analytical balance (Mettler Toledo PB 302^®^, Switzerland). At the end of the post-infection monitoring, isolates were categorised as acute infection causing (mean survival time [MST] ≤ 10 days), subacute infection causing (MST > 10 days but < 30 days) and chronic infection causing (MST ≥ 30 days).

#### Tsetse infection and transmissibility studies

The 0- to 3-day-old teneral male *G. pallidipes* flies were divided into 10 experimental groups, each consisting of 100 flies except one group, which had 50 flies, for infection with the five *T. congolense* and five *T. brucei* spp. isolates. To initiate the infections, tsetse flies were allowed to feed on infected donor Swiss white mice at 25 flies per mouse; the mice were presented to the flies while at peak parasitaemia of antilog 8.4 (Herbert & Lumsden 1976). The flies that successfully fed on the infective blood meal were thereafter maintained on clean (uninfected) bovine blood diet fed in vitro on alternate days as previously described (Feldmann 1994). Flies that failed to feed were excluded from the experiment. A control group of 100 uninfected flies were also caged in groups of 25 flies and maintained *in vitro* on a bovine clean blood diet throughout the study period of 30 days. Fly mortalities for both the infected and uninfected flies were recorded every alternate day for the entire monitoring period, and the difference in mortality was compared. Flies surviving at 30 days post-infection (DPI) were subjected to xenodiagnoses to detect those with mature trypanosome infections as described by Thuita et al. (2008). Thereafter, the surviving flies were dissected using the standard method described by Lloyd (1924). The proboscis, hypopharynx, labrum, salivary glands and midgut were separately suspended in physiological saline and examined for the presence of parasites.

### Statistical analysis

Data were managed using Microsoft Excel (Microsoft USA, version 2010). A general linear model in Genstat 14 was employed to analyse the data. The mean parasitaemia, PCV and body weight data were considered as the response variables and used to compare the different groups over DPI. Cumulative tsetse fly mortality over the 30-day monitoring period and trypanosome counts in various tsetse organs were compared only using proportions. Mouse survival data were analysed by Kaplan–Meier method for determination of survival distribution function. Rank tests of homogeneity were used to determine the effect of trypanosome infection on survival times.

## Results

### Pre-patent period and parasitaemia progression

Four of the five *T. congolense* isolates were all confirmed to be *T. congolense* savannah and one of five as *T. congolense kilifi* by polymerase chain reaction (PCR) (Joanna Auma, unpublished data). The four *T. congolense* savannah produced patent parasitaemia in mice while the remaining isolate, a *T. congolense kilifi* KETRI 2784, did not produce patent parasitaemia and its virulence was therefore not determined (Table 2). The four *T. congolense* savannah isolates had PP ranging from mean ± standard error (SE) values of 4 days for isolate KETRI 2909 to 11.2 ± 2.4 for KETRI 2773 (Table 2); the mean PP differed significantly (*p* < 0.05). When all the mice infected with *T. congolense* isolates were considered as a single group, the PP was a mean ± SE value of 8.4 ± 0.9 (range, 4–11) days. Within the *T. brucei* group, all five isolates produced patent parasitaemia, with comparatively short PP ranging from mean ± SE values of 3.7 ± 0.2 (EATRO 2225) to 6 ± 0.2 days (EATRO 1579) as shown in Table 2; the mean PP of the five *T. brucei* isolates did not differ significantly (*p* > 0.05). When considered as a single group, the *T. brucei* isolates had a mean ± SE PP of 4.5 ± 0.2 (range, 4–6 days).

**TABLE 2:** Variations in pre-patent, survival times and terminal parasitaemia of mice infected with *Trypanosoma congolense* and *Trypanosoma brucei* isolates.

Species	Stabilate number	PP (mean ± SEM)	Survival times (mean ± SEM)	Terminal parasitaemia	Virulence status
*Trypanosoma congolense* savannah	EATRO 1829	9.6 ± 1.6	13.2 ± 1.2	1.0 × 10^9^/mL	Subacute
	KETRI 2773	11.2 ± 2.4	20 ± 4.1	(one peak)	Subacute
	KETRI 2909	4 ± 0	9 ± 2.2	1.0 × 10^9^/mL	Acute
	EATRO 2254	9.6 ± 0.4	28.2 ± 1.2	1.2 × 10^6^/mL	Subacute
*Trypanosoma congolense kilifi*	KETRI 2784	N/D	**-**	-	**-**
*Trypanosoma brucei*	EATRO 2225	3.7 ± 0.2	≥ 30	1.0 × 10^9^/mL	Chronic
	EATRO 2267	3.7 ± 0.5	≥ 30	2.2 × 10^8^/mL	Chronic
	KETRI 2795	4 ± 0	≥ 30	6.3 × 10^7^/mL	Chronic
	EATRO 1579	6 ± 0.2	20 ± 1.3	1.0 × 10^9^/mL	Subacute
	EATRO 1784	4.6 ± 0.7	20 ± 2.4	1.0 × 10^9^/mL	Subacute

N/D, not determined; PP, pre-patent; SEM, mean standard error.

Parasitaemia profiles of the four *T. congolense* savannah isolates exhibited wide variations. Isolate KETRI 2909 attained peak parasitaemia of 1× 10^9^ trypanosomes/mL of blood by 6 DPI and showed only minimal fluctuation throughout the observation period (Figure 1a). In contrast, the parasitaemia induced by the remaining isolates, EATRO 1829, 2254 and 2773, increased gradually with more pronounced fluctuations in peak parasitaemia of successive waves (Figure 1a). Notably, terminal parasitaemia attained high levels of 1× 10^9^ trypanosomes/mL of blood except in isolate KETRI 2254 (Table 2 and Figure 1a). In mice infected with the five *T. brucei* spp. isolates, the parasitaemia patterns were characterised by a rapid increase and attainment of peak levels within 6–8 DPI; this first peak was well controlled in all the isolates (Figure 1b). In contrast, the second wave of high parasitaemia was sustained throughout the experiment (Figure 1b) with terminal parasitaemia reaching 1× 10^9^ trypanosomes/mL for three of five isolates (Table 2). Parasitaemia levels were significantly higher (*p* < 0.01) in *T. brucei*– than in *T. congolense*–infected mice (Figure 1-A1).

**FIGURE 1:**
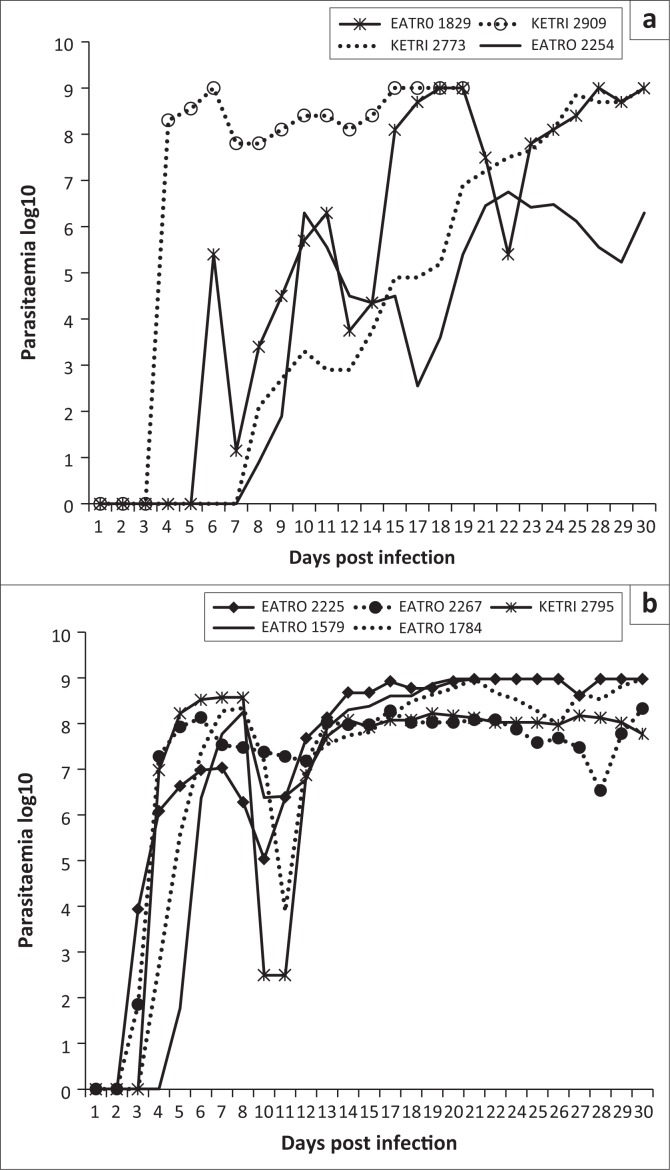
Parasitaemia patterns in mice infected with (a) *Trypanosoma congolense* isolates and (b) *Trypanosoma brucei* isolates.

### Packed cell volume

**FIGURE 2:**
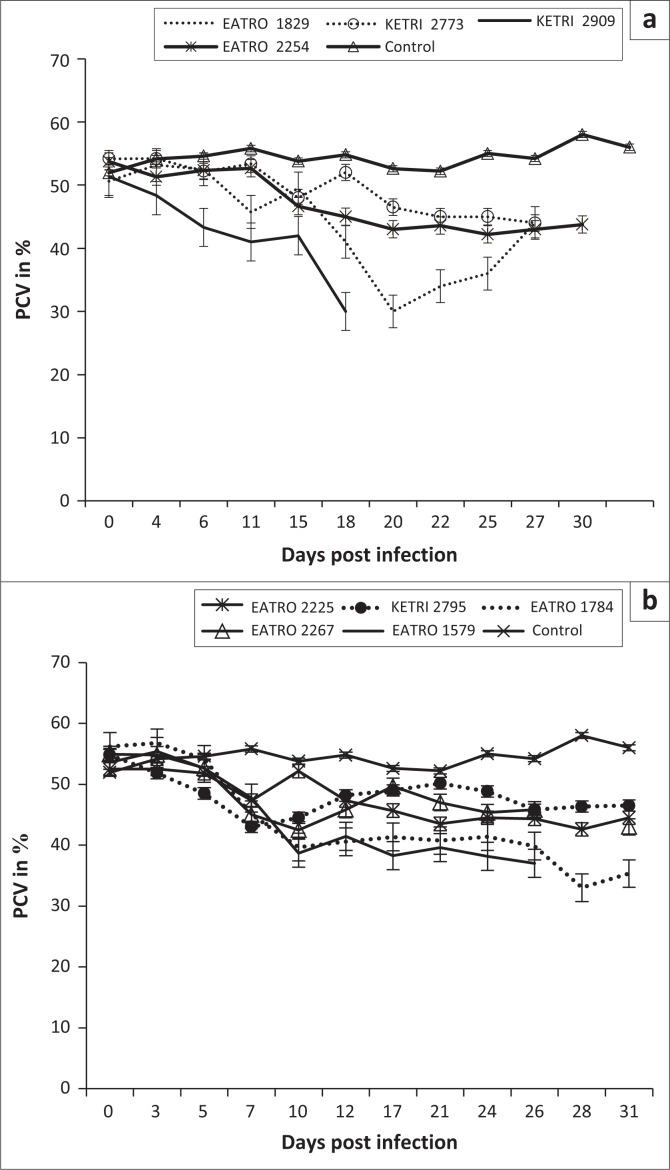
Mean packed cell volume profiles of mice (a) (*n* = 5) infected with isolates of *Trypanosoma congolense* savannah and (b) (*n* = 6) infected with various isolates of *Trypanosoma brucei*.

Animals in the control group had a PCV value of mean ± SE of 51.9 ± 0.6 at day 0; their PCV increased significantly (*p* < 0.001) to mean ± SE of 58 ± 0.6 during the observation period of 30 days (Figure 2a). In contrast, a decline in PCV values was observed in all mice infected with either *T. congolense* or *T. brucei* isolates when compared with their pre-infection baseline values as well as the control mice (Figures 2a and b), indicating occurrence of trypanosome-induced anaemia in all experimental groups. However, with the exception of isolate *T. congolense* KETRI 2909 for which the PCV declined by 16% by 7 DPI (Figure 2a), the anaemia in mice groups infected with the remaining three isolates had a slow onset and was only observed at 21 DPI when the mice had lost an average of 24% (range, 14.2% – 40.7%) of their pre-infection baseline PCV values (Figure 2a). Thereafter, the PCV of *T. congolense*–infected mice stabilised (isolates EATRO 2254 and KETRI 2773) and recovered moderately (EATRO 1829) as shown in Figure 2a. In contrast to the pattern in *T. congolense*–infected mice, all the mice groups that were infected with *T. brucei* isolates (Figure 2b), experienced a rapid onset in PCV declines. At 7 DPI, all five *T. brucei*–infected mice groups exhibited PCV declines ranging from 9.9% (EATRO 2225) to 22% (EATRO 2795) as shown in Figure 2b; these differences between isolates were statistically significant (*p* < 0.05). Thereafter, at 7–14 DPI, the PCV of all infected mice groups stabilised (Figure 2b). Terminally, mice groups infected with isolates EATRO 1579 and EATRO 1784 exhibited the greatest PCV declines of 26% and 41%, respectively.

### Body weight changes

**FIGURE 3:**
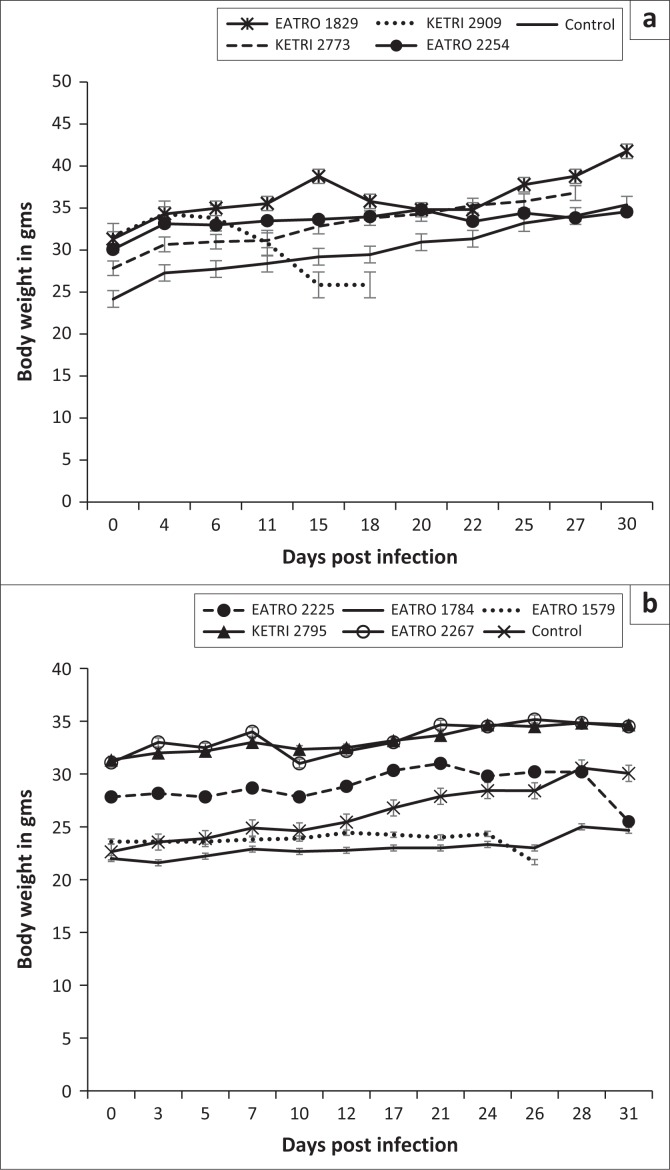
Bodyweight profiles of mice (a) (mean ± standard error of the mean; *n* = 4) infected with various isolates of *Trypanosoma congolense* and (b) (mean ± standard error of the mean; *n* = 5) infected with various isolates of *Trypanosoma brucei*.

The baseline body weights of all mice groups ranged from mean ± SE of 22 ± 0.8 to 31.5 ± 0.9. Infection of mice with *T. congolense* KETRI 2909 induced a decline in body weight from mean ± SE of 31.8 g ± 0.7 g at day 0 to mean ± SE of 26.0 g ± 0 g at 14 DPI equivalent to a 18.2% drop (Figure 3a). However, body weight of mice infected with the remaining three *T. congolense* isolates, EATRO 2254, 1829 and KETRI 2773, was unaffected by infection as shown by an increase of 11% – 23.2% in comparison with their respective baseline values and in a similar pattern with control mice (Figure 3a). In the groups of mice infected with *T. brucei*, the bodyweight profiles were largely comparable to those observed in the control group of mice (Figure 3b). However, mice groups infected with two of the isolates, EATRO 1579 and EATRO 2225, exhibited a terminal decline in body weight (Figure 3b).

### Survival time

**FIGURE 4:**
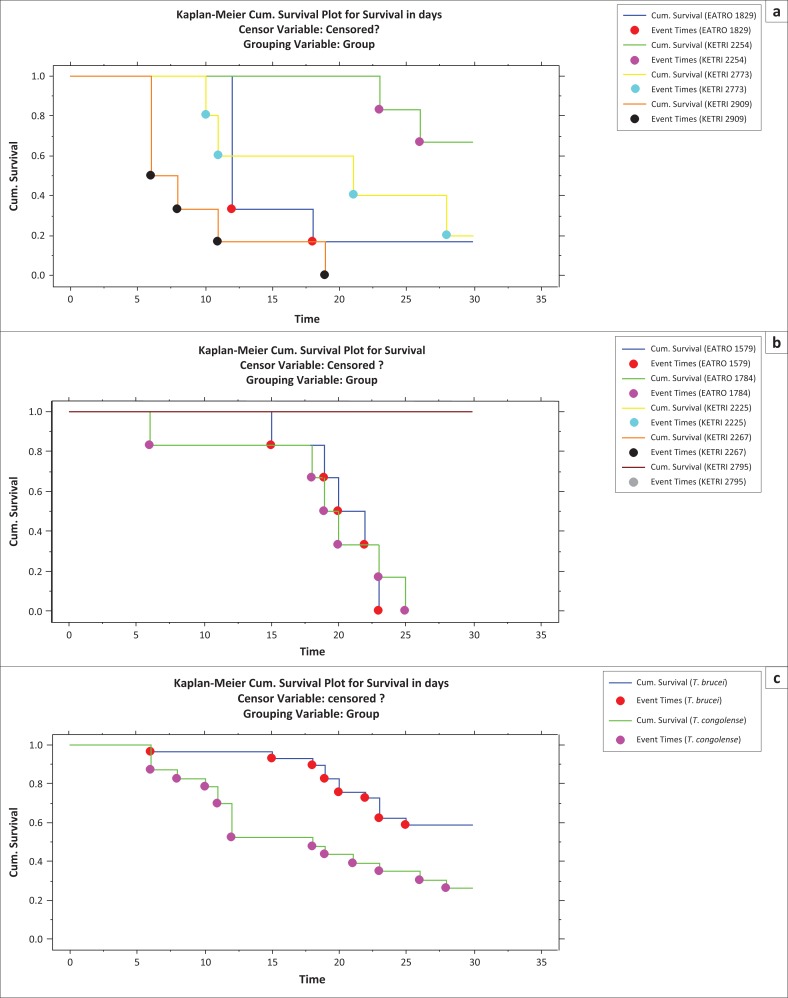
Survival distribution functions of mice infected with various (a) *Trypanosoma congolense* isolates, (b) *Trypanosoma brucei* isolates and (c) *Trypanosoma brucei* and *Trypanosoma congolense* trypanosome isolates.

All control mice survived up to the end of the experimental period of 30 days and their survival time data were therefore categorised as censored. Mice infected with *T. congolense* had a MST of between 9 ± 2.1 (KETRI 2909) and 28 ± 1.2 (EATRO 2254) days as shown (Table 2). These differences were significant as demonstrated by the *p*-value associated with Wilcoxon of 0.0005 and log rank of 0.0004 (Table 2, Figure 4a). Mice infected with three of five *T. brucei* group of trypanosomes (EATRO 2225, EATRO 2267 and KETRI 2795) all survived beyond 30 days. However, the MST for mice infected with EATRO 1579 and 1784 were 20.3 ± 1.3 and 20 ± 2.4 days, respectively (Table 2); these MSTs were not statistically different (Wilcoxon *p* = 0.21, log rank *p* = 0.07). Overall, the MST of all *T. brucei*–infected mice was longer than that of *T. congolense*–infected mice. The *p*-value associated with rank tests of homogeneity were both significant with that for Wilcoxon test (0.002) being smaller than that for log rank test (0.005). This suggested that the two species differed primarily at early survival time with significantly (*p* < 0.001) longer early survival time in *T. brucei*–infected mice compared with those infected with *T. congolense* (Figure 4b).

### Tsetse survival time and trypanosome transmissibility results

At the 30-day time point, a cumulative 37% mortality was recorded in the control group of flies. However, in the infected fly groups, the mortality rates ranged from 59% to 70% for *T. congolense* and from 35% to 74% for the *T. brucei* tsetse fly groups (Table 3). The highest tsetse mortality (74%) was recorded in flies fed on *T. brucei* EATRO 2267–infected mice. Xenodiagnosis of the tsetse flies surviving at 30 DPI showed that only one of five *T. congolense* isolates, EATRO 1829, successfully transmitted infection to clean mice, with a total of 14 successful fly transmissions. Upon subsequent dissection and microscopic examination of the infection status of all 39 surviving tsetse flies in this group, it was determined that 15/39 (38.5%) flies had mature mouthpart infections (Table 3) meaning that the transmission efficacy for EATRO 1829–infected flies was 14/15 (93%) as shown in Table 3. Tsetse flies infected with two isolates, KETRI 2784 the only *T. congolense kilifi* among the test isolates and *T. congolense* savannah and KETRI 2254, did not transmit any infections to mice despite having mature infection rates of 9.8% and 10%, respectively (Table 3).

**TABLE 3:** Tsetse infection, mortality and transmissibility rates.

Stabilate number and species of trypanosome	Total tsetse flies infected	Total mortality		Midgut infection		Mature infection		Successful transmission to clean mice	
		*n*	%	*n*	%	*n*	%	*n*	%
***Trypanosoma congolense* isolates**									
KETRI 2909, savannah	100	59	-	1/41	2.4	0	-	0	-
KETRI 2773, savannah	100	48	-	0	-	0	-	0	-
EATRO 2254, savannah	100	70	-	3/30	10.0	3/30	10.0	0	-
EATRO 1829, savannah	100	61	-	15/39	38.5	15/39	38.5	14/15	93.3
KETRI 2784, *kilifi*	100	59	-	3/41	7.3	4/41	9.8	0	**-**
**-**	**-**	297	59.4	**-**	**-**	**-**	**-**	**-**	**-**
***Trypanosoma brucei* isolates**									
EATRO 2267	100	74	-	6/26	23.1	6/26	23.1	6/6	100
EATRO 2225	50	36	-	0	-	0		0	**-**
KETRI 2795	100	58	-	1/42	2.4	1/42	2.4	1/1	100
EATRO 1579	100	44	-	2/56	3.6	1/56	1.8	†	**-**
EATRO 1784	100	35	-	1/65	1.5	4/65	6.2	†	**-**
**-**	**-**	247	54.9	-	-	**-**	**-**	**-**	**-**
**Control**	100	37	-	-	-	-	-	-	-

† Not submitted to transmission infection experiment.

Microscopic examination of flies exposed to infective blood meals containing *T. brucei* isolates revealed that tsetse flies infected with *T. brucei* EATRO 2267 had the highest mature infection rates of 6/26 (23.1%) and a complete transmission efficacy of 6/6 (100%) as shown in Table 3. The only other *T. brucei* isolate that had mature salivary gland and/or mouthparts infection, KETRI 2795, also demonstrated a transmission efficacy of 1/1 (100%) as shown (Table 3). EATRO 1579 and 1784 exhibited mature salivary gland or mouthparts and immature midgut infections but were not evaluated for transmission efficacy. One of the five isolates, EATRO 2225, did not show any immature or mature infections (Table 3).

## Ethical considerations

All protocols and procedures used in this study involving laboratory animals were reviewed and approved by the Institutional Animal Care and Use Committee of KALRO-BioRI.

## Discussion

In this study, we have characterised the virulence and tsetse transmissibility patterns of five *T. congolense* and *T. brucei* spp. isolates currently preserved at the KALRO cryobank. The work was conceived to ensure availability of well-characterised biological materials for research by the scientific community as previously reported in Murilla et al. (2014) and generates insights into the dynamics of *T. congolense* and *T. brucei* caused AAT through studies in the murine model. A total of 4/5 (80%) of the *T. congolense* isolates were infective to mice, which is evidence of the utility of the mouse model for infectivity and pathogenicity studies of this species of trypanosomes (Magez & Caljon 2011). However, *T. congolense* KETRI 2784 was not infective to the experimental group (*n* = 6) of mice despite being shown to have motile trypanosomes on wet smear and being infective to immunosuppressed donor mice (KALRO-BioRI, unpublished data). PCR results identified *T. congolense* KETRI 2784 isolate to be a *T. congolense kilifi* unlike the other four of five isolates, which were *T. congolense* savannah (Joanna Auma, unpublished data). The failure of this isolate to grow in mice with an intact immune system is probably an indicator of its low virulence, consistent with previous reports that *T. congolense* savannah were more virulent than *T. congolense kilifi* (Bengaly et al. 2002; Motloang et al. 2014). All 5/5 (100%) *T. brucei* spp. isolates were infectious to mice, and all were negative for the serum-resistant associated gene, thus confirming their identity as *T. brucei*.

We used MST of mice post-infection as the main indicator of virulence. On this basis, there were distinct differences in virulence among the isolates, with MST ranging from 9 to 28 days for *T. congolense* isolates and from 20 to more than 30 days for *T. brucei isolates*. Survival time of infected mice has previously demonstrated wide variations in virulence among *T. congolense* isolates recovered from AAT foci in Zambia (Masumu et al. 2006; Van den Bossche et al. 2011) and KwaZulu-Natal in the Republic of South Africa (Motloang et al. 2014). In their studies, these authors further reported that the proportion of highly virulent parasites was greater in isolates recovered from wild animals (sylvatic cycle) than in isolates recovered from domestic animals (Motloang et al. 2014; Van den Bossche et al. 2011). In our current study, two of four *T. congolense* savannah isolates, including the highly virulent *T. congolense* KETRI 2909, were recovered from cattle in the Galana area of the Kenyan Coast region (Table 1), an area where the likelihood of transmission of wildlife derived isolates to livestock is significant. Therefore, it is possible that our finding that *T. congolense* isolates were in general characterised by shorter MST than *T. brucei* isolates could be because of the recent transmission from the sylvatic cycle. Importantly, our results confirm the occurrence of intra- and inter-species differences in virulence among trypanosome isolates, suggesting that these differences may play a significant role in the severity of clinical profiles in trypanosomiasis. Such parasite virulence factors need to be comprehensively elucidated as suggested by Morrison (2011).

In addition to MST, we used parasitaemia and clinical parameters to compare variation in virulence among the study isolates. Our results indicated that there were statistically significant (*p* < 0.05) differences in the mean PP of *T. congolense* isolates but not for *T. brucei* isolates. Importantly, the *T. congolense* isolate that had the shortest PP of 4 days also had the shortest MST, showing that PP has value as an indicator of virulence. However, the full PP data (Table 2) also show that *T. brucei* isolates with relatively short pp periods (≤ 4 days) ended up causing chronic infection (≥ 30 days of ST), suggesting that as a whole, PP should not be used in isolation to categorise the virulence status of trypanosomes. The wide variations in PP of *T. congolense* isolates were in agreement with previous reports (Bengaly et al. 2002; Masumu et al. 2006). After the parasitaemia became patent, mice infected with *T. brucei* isolates were in general characterised by significantly (*p* < 0.01) higher parasitaemia loads than the *T. congolense* isolates causing more acute infection on the basis of MST. The differences in parasitaemia profiles of the individual isolates (Figures 1a and b) and the overall mean parasitaemia of *T. congolense* and *T. brucei* isolates (Figure 1-A1) indicated that as with PP, the use of parasitaemia loads to compare virulence status of isolates is best restricted to isolates of the same species. Our study did not explore the reasons for the generally lower parasitaemia profiles of *T. congolense* as compared to *T. brucei* spp. However, previous studies have demonstrated the release of trypanotoxic nitric oxide (NO) by macrophage cells of animals infected with the two species of trypanosome isolates (Wenfa et al. 2011). While NO contributes to the control of *T. congolense* parasitaemia (Wenfa et al. 2011), it does not contribute to control of *T. brucei* parasitaemia in vivo (Hertz & Mansfield 1999).

In contrast with *T. brucei* isolates, which exhibited only two prominent parasitaemic waves, the parasitaemia patterns of *T. congolense* isolates were characterised by more frequent but short-duration parasitaemic waves (Figures 1a and b), suggesting occurrence of frequent changes in the dominant variable antigenic types (VAT) that may lead to the exhaustion of the host immune system. This could explain why mice infected with *T. congolense* isolates had, in general, shorter survival times than *T. brucei*–infected mice in the current study and perhaps also explains the difference in the disease severity observed in the field (Biryomumaisho & Katunguka-Rwakishaya 2007). Variations in virulence in isolates of the same species has previously been attributed to sexual recombination (mating), thus enhancing genetic diversity even within a population from a single endemic focus (MacLeod, Tait & Turner 2001; Morrison et al. 2009).

Anaemia and weight loss were key features in mice groups infected with the *T. congolense* and *T. brucei* isolates as shown in Figures 2a and b, a result that is in agreement with previous observations in trypanosome infections in mice (Doko et al. 1997; Ikede, Lule & Terry 1977) and other laboratory animals (Thuita et al. 2008). In general, the onset of anaemia was slower in *T. congolense*– than in *T. brucei*–infected mice groups, which can be attributed to the lag time before development of patent infections in mice infected with the two species of trypanosomes. In mice groups infected with *T. congolense* KETRI 2909, onset of anaemia was most rapid, consistent with its status as the most virulent *T. congolense* isolate. In agreement with our findings, a previous study involving injection of mice with virulent and less virulent *T. congolense* strains reported that the drop in PCV was significantly more in the virulent than in the less virulent strains (Masumu et al. 2009), suggesting that PCV is a reliable marker of the degree of virulence of an isolate. Despite the fact that both species of trypanosmes cause anaemia, results from a previous study show that the mechanisms underlying anaemia may be different between trypanosome species, with evidence for tumour necrosis factor-α being involved in anaemia induced by *T. brucei* spp. infection but not in anaemia induced by *T. congolense* infection (Naessens et al. 2005). Body weight results showed that other than *T. congolense* KETRI 2909, which induced a significant decline in body weight of the infected mice, body weight changes in mice groups that were infected with the other three *T. congolense* isolates, EATRO 2254, 1829 and KETRI 2773, and all five *T. brucei* isolates exhibited a similar pattern with the uninfected control group of mice (Figures 3a and b), indicating that body weight was not adversely affected by infection during the 30 days of post-infection monitoring observed in this study. While the slight increase in body weight of *T. congolense*–infected mice is in agreement with a previous observation (Noyes et al. 2009), it is not clear why the body weight is not affected by the infection, but it is suggestive that body weight alone cannot reliably be used in determining pathogenicity using Swiss white mice.

The *T. congolense* isolate KETRI 2909 that was found to be the most virulent in the murine pathogenicity study, had a midgut infection rate of 2.4% and zero mature infections, suggesting that it had a reduced chance of being transmitted. In contrast, the *T. congolense* isolate EATRO 1829 which caused subacute infection had a mature infection of 38% indicating better prospects of being transmitted. Similarly, one of the *T. brucei* isolates EATRO 2267 that caused chronic infection had the highest proportions of mature trypanosome infection of 23% and a transmission efficacy of 6/6 (100%), indicating an improved chance of being transmitted. However, not all isolates causing subacute and chronic infections were equally well transmitted by flies (Table 3), suggesting that this property is isolate dependent. In addition, our study found that the proportion of *T. congolense*–infected tsetse flies with infection in the midgut (immature) and mouthparts (mature) was greater than the comparable data for *T. brucei*–infected flies, which was in agreement with a previous study on susceptibility of tsetse flies to *T. congolense* and *T. brucei* trypanosomes (Peacock et al. 2012). This indicates that vector comptence could be a factor for the observed higher prevalence of bovine *T. congolense* infections as compared with *T. brueci* infection (Desta et al. 2013; Majekodunmi et al. 2013). However, for a majority of the isolates, the proportion of flies with mature infections was less than 10%, which can be attributed to the effectiveness of the tsetse intrinsic defence mechanisms against trypanosome infection migration from foregut to salivary glands and/or mouthparts as previously observed (Gibson & Bailey 2003; Peacock et al. 2012).

## Conclusion

In conclusion, results from the current study confirm the occurrence of differences in virulence among *T. congolense* and *T. brucei* isolates from the same eastern African region. The transmissibility of these isolates by *G. pallidipes* was most efficient for one subacute infection–causing isolate, *T. congolense* EATRO 1829, and one chronic infection–causing isolate, *T. brucei* EATRO 2267, which is contrary to an earlier observation associating virulence with high levels of vector infection rates and rapid transmission (Masumu et al. 2006). We further identified *T. congolense* EATRO 1829 and *T. brucei* EATRO 2267 as suitable for tsetse infectivity and transmissibility experiments.

## Figures and Tables

**FIGURE 1-A1:**
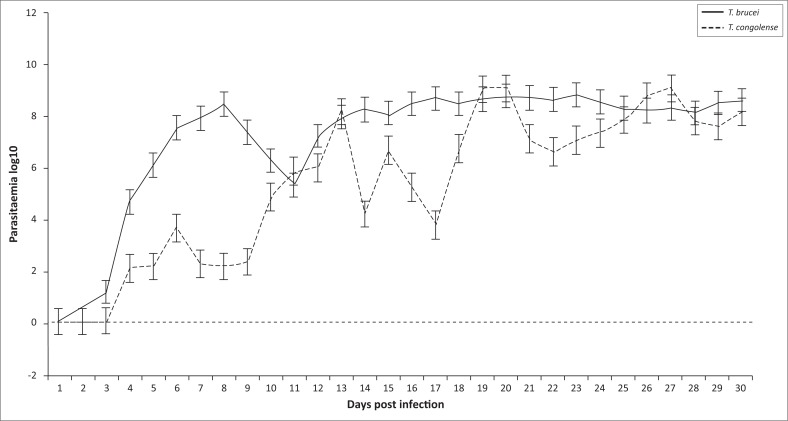
Comparison of mean ± standard error parasitaemia profiles in mice infected with *Trypanosoma brucei* and *Trypanosoma congolense* isolates.

## References

[CIT0001] Bengaly Z., Sidibe I., Boly H., Sawadogo L. & Desquesnes M, 2002, ‘Comparative pathogenicity of three genetically distinct *Trypanosoma congolense*-types in inbred Balb/c mice’, *Veterinary Parasitology* 105, 111–118. https://doi.org/10.1016/S0304-4017(01)00609-4 11900925 10.1016/s0304-4017(01)00609-4

[CIT0002] Biryomumaisho S. & Katunguka-Rwakishaya E, 2007, ‘The pathogenesis of anaemia in goats experimentally infected with *Trypanosoma congolense* or *Trypanosoma brucei*: Use of the myeloid:erythroid ratio’, *Veterinary Parasitology* 143, 354357 https://doi.org/10.1016/j.vetpar.2006.08.030 10.1016/j.vetpar.2006.08.030 16982150

[CIT0003] Brun R., Hecker H. & Lun Z, 1998, ‘Trypanosoma evansi and T. equiperdum: Distribution, biology, treatment and phylogenetic relationship (a review)’, *Veterinary Parasitology* 79, 95–107. https://doi.org/10.1016/S0304-4017(98)00146-0 9806490 10.1016/s0304-4017(98)00146-0

[CIT0004] Ciosi M., Masiga D. & Cmr T, 2014, ‘Laboratory colonisation and genetic bottlenecks in the tsetse fly glossina pallidipes’, *PLOS Neglected Tropical Diseases* 8, e2697 https://doi.org/10.1371/journal.pntd.0002697 24551260 10.1371/journal.pntd.0002697 PMC3923722

[CIT0005] David B.J. & McCulloch R, 2001, ‘Antigenic variation in trypanosomes: Enhanced phenotypic variation in a eukaryotic parasite’, *Advances in Parasitology* 49, 2–70. 10.1016/s0065-308x(01)49037-3 11461029

[CIT0006] Desquesnes M. & Dia M.L, 2004, ‘Mechanical transmission of *Trypanosoma vivax* in cattle by the African tabanid Atylotus fuscipes’, *Veterinary Parasitology* 119, 9–19. https://doi.org/10.1016/j.vetpar.2003.10.015 15036572 10.1016/j.vetpar.2003.10.015

[CIT0007] Desquesnes M., Holzmuller P., Lai D.-H., Dargantes A., Lun Z.-R. & Jittaplapong S, 2013, ‘Trypanosoma evansi and Surra: A review and perspectives on origin, history, distribution, taxonomy, morphology, hosts, and pathogenic effects’, *BioMed Research International* 2013, 194176. 24024184 10.1155/2013/194176 PMC3760267

[CIT0008] Desta M., Beyene D. & Haile S, 2013, ‘Trypanosome infection rate of Glossina pallidipes and trypanosomosis prevalence in cattle in Amaro Special District of Southern Ethiopia’, *Journal of Veterinary Medicine and Animal Health* 5, 164–170.

[CIT0009] Doko A., Verhulst A., Pandey V.S., van der Stuyft P, 1997, ‘Artificially induced Trypanosoma brucei brucei infection in Lagune and Borgou cattle in Benin’, *Veterinary Parasitology* 69, 151–157. https://doi.org/10.1016/S0304-4017(96)01097-7 9187040 10.1016/s0304-4017(96)01097-7

[CIT0010] Duffy C.W., Maclean L., Sweeney L., Cooper A., Turner C.M.R., Tait A. et al., 2013, ‘Population genetics of *Trypanosoma brucei* rhodesiense: Clonality and diversity within and between Foci’, *PLOS Neglected Tropical Diseases* 7, e2526 https://doi.org/10.1371/journal.pntd.0002526 24244771 10.1371/journal.pntd.0002526 PMC3828156

[CIT0011] Feldmann H, 1994, *Guidelines for the rearing of tsetse flies using the membrane feeding technique*, ICIPE Science Press, Nairobi, Kenya.

[CIT0012] Gibson W. & Bailey M, 2003, ‘The development of *Trypanosoma brucei* within the tsetse fly midgut observed using green fluorescent trypanosomes’, *Kinetoplastid Biology and Disease* 2, 1. 12769824 10.1186/1475-9292-2-1 PMC156611

[CIT0013] Herbert W. & Lumsden W, 1976, ‘*Trypanosoma brucei*: A rapid “Matching” method for estimating the host’s parasitaemia’, *Experimental Parasitology* 40, 427–431. https://doi.org/10.1016/0014-4894(76)90110-7 976425 10.1016/0014-4894(76)90110-7

[CIT0014] Hertz C.J. & Mansfield J.M, 1999, ‘IFN-γ-dependent nitric oxide production is not linked to resistance in experimental African trypanosomiasis’, *Cellular Immunology* 192, 24–32. https://doi.org/10.1006/cimm.1998.1429 10066343 10.1006/cimm.1998.1429

[CIT0015] Ikede B., Lule M. & Terry R, 1977, ‘Anaemia in trypanosomiasis: Mechanisms of erythrocyte destruction in mice infected with Trypanosoma congolense or T. brucei’, *Acta Tropica* 34, 53–60. 16466

[CIT0016] Ilemobade A, 2009, ‘Tsetse and trypanosomosis in Africa: The challenges, the opportunities’, *The Onderstepoort Journal of Veterinary Research* 76, 35–40. https://doi.org/10.4102/ojvr.v76i1.59 19967926 10.4102/ojvr.v76i1.59

[CIT0017] Lloyd L., Johnson W.B., Young W.A. & Morrison H, 1924, ‘Second report of the tsetse fly investigations in the northern provinces of Nigeria’, *Bulletin of Entomological Research* 15, 1–17. https://doi.org/10.1017/S000748530004606X

[CIT0018] Macleod A., Tait A. & Turner C, 2001, ‘The population genetics of Trypanosoma brucei and the origin of human infectivity’, *Philosophical Transactions of the Royal Society of London. Series B, Biological Sciences* 356, 1035–1044. https://doi.org/10.1098/rstb.2001.0892 11516381 10.1098/rstb.2001.0892 PMC1088498

[CIT0019] Magez S. & Caljon G, 2011, ‘Mouse models for pathogenic African trypanosomes: Unravelling the immunology of host-parasite-vector interactions’, *Parasite Immunology* 33, 423–429. https://doi.org/10.1111/j.1365-3024.2011.01293.x 21480934 10.1111/j.1365-3024.2011.01293.x

[CIT0020] Majekodunmi A.O., Fajinmi A., Dongkum C., Picozzi K., Thrusfield M.V. & Welburn S.C, 2013, ‘A longitudinal survey of African animal trypanosomiasis in domestic cattle on the Jos Plateau, Nigeria: Prevalence, distribution and risk factors’, *Parasit Vectors* 6, 239 https://doi.org/10.1186/1756-3305-6-239 23958205 10.1186/1756-3305-6-239 PMC3765779

[CIT0021] Masumu J., Marcotty T., Geertsb S., Vercruyssec J. & Van den Bossche P, 2009, ‘Cross-protection between Trypanosoma congolense strains of low and high virulence’, *Veterinary Parasitology* 163, 127–131. https://doi.org/10.1016/j.vetpar.2009.04.006 19423225 10.1016/j.vetpar.2009.04.006 PMC2771272

[CIT0022] Masumu J., Marcotty T., Ndeledje N., Kubi C., Geerts S., Vercruysse J. et al., 2006, ‘Comparison of the transmissibility of Trypanosoma congolense strains, isolated in a trypanosomiasis endemic area of eastern Zambia, by Glossina morsitans morsitans’, *Parasitology* 133, 331–334. https://doi.org/10.1017/S0031182006000369 16719960 10.1017/S0031182006000369

[CIT0023] Matthews K.R, 2005, ‘The developmental cell biology of *Trypanosoma brucei*’, *Journal of Cell Science* 15, 283–290. https://doi.org/10.1242/jcs.01649 10.1242/jcs.01649 PMC2686837 15654017

[CIT0024] Morrison L, 2011, ‘Parasite-driven pathogenesis in *Trypanosoma brucei* infections’, *Parasite Immunology* 33, 448–455. https://doi.org/10.1111/j.1365-3024.2011.01286.x 21366624 10.1111/j.1365-3024.2011.01286.x PMC3443366

[CIT0025] Morrison L., Tweedie A., Black A., Pinchbeck G., Christley R., Schoenefeld A. et al., 2009, ‘Discovery of mating in the major African livestock pathogen *Trypanosoma congolense*’, *PLoS One* 4, e5564 https://doi.org/10.1371/journal.pone.0005564 19440370 10.1371/journal.pone.0005564 PMC2679202

[CIT0026] Motloang M., Masumu J., Mans B.J. & Latif A.A, 2014, ‘Virulence of *Trypanosoma congolense* strains isolated from cattle and African buffaloes (Syncerus caffer) in KwaZulu-Natal, South Africa’, *The Onderstepoort Journal of Veterinary Research* 81, 1–7. https://doi.org/10.4102/ojvr.v81i1.679 10.4102/ojvr.v81i1.679 25685920

[CIT0027] Murilla G., Ndung’u K., Thuita J., Gitonga P., Kahiga D., Auma J. et al., 2014, ‘Kenya Trypanosomiasis Research Institute Cryobank for Human and Animal trypanosomes isolates to support Research: Opportunities and challenges’, *PLOS Neglected Tropical Diseases* 8, e2747 https://doi.org/10.1371/journal.pntd.0002747 24853062 10.1371/journal.pntd.0002747 PMC4031132

[CIT0028] Naessens J., Kitani H., Nakamura Y., Yagi Y., Sekikawa K. & Iraqi F, 2005, ‘TNF-alpha mediates the development of anaemia in a murineTrypanosoma brucei rhodesiense infection, but not the anaemia associated with a murine Trypanosoma congolense infection’, *Clinical and Experimental Immunology* 139, 405–410. https://doi.org/10.1111/j.1365-2249.2004.02717.x 15730385 10.1111/j.1365-2249.2004.02717.x PMC1809320

[CIT0029] Noyes H., Alimohammadian M., Agaba M., Brass A, Fuchs H. & Gailus-Durner V, 2009, ‘Mechanisms controlling anaemia in Trypanosoma congolense infected mice’, *PLoS One* 4, e5170 https://doi.org/10.1371/journal.pone.0005170 19365556 10.1371/journal.pone.0005170 PMC2664899

[CIT0030] Peacock L., Cook S., Ferris V., Bailey M. & Gibson W, 2012, ‘The life cycle of Trypanosoma (Nannomonas) congolense in the tsetse fly’, *Parasit Vectors* 5, 109 https://doi.org/10.1186/1756-3305-5-109 22676292 10.1186/1756-3305-5-109 PMC3384477

[CIT0031] Stephen L. (ed.), 1986, *Trypanosomiasis, a veterinary perspective*, Pergamon Press, Oxford.

[CIT0032] Stijlemans B., Caljon G., Abbeele J.V.D., Ginderachter J.A.V., Magez S. & Trez C.D, 2016, ‘Immune evasion strategies of Trypanosoma brucei within the mammalian host: Progression to pathogenicity’, *Frontiers in Immunology* 7, 233 https://doi.org/10.3389/fimmu.2016.00233 27446070 10.3389/fimmu.2016.00233 PMC4919330

[CIT0033] Thuita J., Kagira J., Mwangangi D., Matovu E., Turner C. & Masiga D, 2008, ‘Trypanosoma brucei rhodesiense transmitted by a single tsetse fly bite in vervet monkeys as a model of human African trypanosomiasis’, *PLOS Neglected Tropical Diseases* 2, e238 https://doi.org/10.1371/journal.pntd.0000238 18846231 10.1371/journal.pntd.0000238 PMC2565695

[CIT0034] Van den Bossche P., Chitanga S., Masumu J., Marcotty T. & Delespaux V, 2011, ‘Virulence in Trypanosoma congolense Savannah subgroup. A comparison between strains and transmission cycles’, *Parasite Immunology* 33, 456–460. https://doi.org/10.1111/j.1365-3024.2010.01277.x 21204855 10.1111/j.1365-3024.2010.01277.x

[CIT0035] Wenfa L., Wei G., Pan W. & Tabel H, 2011, ‘Trypanosoma congolense infections: Induced nitric oxide inhibits parasite growth in vivo’, *Journal of Parasitology Research* 2011, 1–10. 10.1155/2011/316067 PMC3092548 21584233

[CIT0036] Wu Y.E., Min F., Pan J., Wang J., Wen Yuan Y.Z., Huang R. & Zhang L, 2015, ‘Systemic Candida parapsilosis Infection Model in Immunosuppressed ICR Mice and Assessing the Antifungal Efficiency of Fluconazole’, *Veterinary Medicine Internationa* 2015, ID 370641, 7 pages. http://dx.doi.org/10.1155/2015/370641 10.1155/2015/370641 PMC4512613 26240775

